# Understanding early inequalities: Multiple dimensions of children's developmental contexts predict age 3 outcomes

**DOI:** 10.1111/bjdp.12569

**Published:** 2025-05-31

**Authors:** Laura A. Outhwaite

**Affiliations:** ^1^ Centre for Education Policy and Equalising Opportunities IOE, UCL's Faculty of Education and Society London UK

**Keywords:** child development, early years, inequalities, socioeconomic circumstances

## Abstract

Inequalities in children's cognitive and socioemotional skills emerge early and persist throughout childhood. This study examines how multiple dimensions of children's developmental contexts, including demographic, socioeconomic and family circumstances, predict age 3 outcomes using data from the UK Household Longitudinal Study (2012–2022). In a cross‐sectional sample of 5700 three‐year‐olds and their families, results showed that child health, the home learning environment, turning 3 during Covid‐19, child ethnicity, parent education and financial strain in the home significantly predicted early outcomes in communication, daily living, socialization and motor skills. Although income‐related eligibility for early years pupil premium did not predict early outcomes, this may reflect the inadequacies of this indicator for capturing all families facing financial difficulties. There was also an increasing gap in early outcomes as children experienced more indicators related to disadvantage, relative to children with no indicators. Overall, this study highlights the importance of a multidimensional approach for understanding and reducing early educational inequalities.

**Statement of Contribution**

**What is already known on this subject?**

- In England, efforts to reduce early inequalities use single indicators, such as EYPP eligibility.
- But other demographic, socioeconomic and family circumstances are also important to consider.

**What does the present study add?**

- Using contemporary data, this study shows children’s developmental contexts are multidimensional.
- EYPP did not predict early outcomes, suggesting it may miss families facing financial difficulties.

## INTRODUCTION

Inequalities in children's cognitive and socioemotional skills are evident from age 3 (Cattan et al., 2024) and persist over time (Tuckett et al., 2024). Addressing these inequalities is a key research, policy and practice target, as early childhood is a crucial period for development with long‐term influences on education, employment and health (Black et al., 2017; Oppenheim & Archer, 2021). In England, approaches to identify and reduce inequalities in children's early outcomes often rely on singular indicators, such as eligibility for Early Years Pupil Premium (EYPP), which is largely determined by parents' receipt of income‐support benefits.^1^ However, recent studies suggest that these income‐based indicators are constrained by narrow eligibility criteria and may not capture the breadth of children's developmental contexts (Campbell et al., 2025).

Evidence highlights the importance of considering multiple dimensions of children's developmental contexts, including demographic, socioeconomic and family circumstances. For example, studies using data from the early 2000s show parental education is a key predictor of children's outcomes (Thornton et al., 2024) as well as other indicators, including ethnicity, family income, cohabitation status, maternal mental health, child health and the home learning environment (Cattan et al., 2024; Dearden et al., 2011). Other studies suggest stressors brought forth by the Covid‐19 pandemic may have exacerbated these early inequalities (Penna et al., 2023). Evidence also shows that socioeconomic indicators about the individual are more predictive of children's outcomes than area‐level indicators (e.g. Income Deprivation Affecting Children Index) (Clery et al., 2022).

When examining how multiple indicators of children's developmental contexts relate to early outcomes, Evans et al. (2013) argue that combining indicators into a single value can provide an efficient way to capture and communicate the complexities of children's experiences. For example, Melhuish and Gardiner (2024) used factor analysis to group indicators into two conceptual categories: an economic factor (family income, parental receipt of benefits), which was linked to poorer language outcomes at age 5, and a home factor (parental mental health, home learning environment, parenting style), which was associated with negative socioemotional outcomes at the same age. However, Thornton et al. (2024) found that multiple indicators considered simultaneously were a better predictor of children's language outcomes at ages 3 and 5 than a factor analysis approach.

Furthermore, factor analysis methods may not account for unique combinations of indicators across factors when examining potential cumulative effects on children's outcomes (Evans et al., 2013). An alternative approach is to use a cumulative effect model. Here, children's experiences of various indicators are dichotomously scored and summed to create a composite developmental context score. However, there is mixed evidence regarding whether this method is more predictive of children's outcomes than considering multiple indicators simultaneously (Evans et al., 2013).

The current study aimed to examine how multiple dimensions of children's developmental contexts are related to early outcomes. The following research questions (RQs) were asked: (1) When considered simultaneously, how are different indicators of children's developmental contexts related to age 3 outcomes? (2) When using a composite developmental context score, what is the cumulative effect of multiple indicators on age 3 outcomes?

## METHODS

### Dataset

The current study used data from Understanding Society, the UK Household Longitudinal Study (UKHLS) (University of Essex, ISER, 2023). UKHLS is a nationally representative probability panel survey of approximately 40,000 households in the United Kingdom (see ISER, 2023). UKHLS contains rich data on child development outcomes and contexts, including indicators of demographic, socioeconomic and family circumstances. UKHLS cross‐sectional weights were used in all reported analyses to account for the clustered and stratified sampling frame. This ensured the findings were nationally representative of the UK population. Ethical approval for this study was granted by the IOE ethics committee (REC 1982).

### Participants

The current study uses cross‐sectional data from households with at least one 3‐year‐old between 2012 (Wave 3) and 2022 (Wave 13). Mothers' responses to the parent indicators were included in the study (Macmillan & Tominey, 2023). Fathers' or other caregivers' responses were included if a mother was not present. The initial sample (*n* = 5810) had a small amount of missing data with a minimal risk of bias (see Supporting Information for how missing data were handled). Table 1 summarizes the final sample of 5700 three‐year‐olds and their caregivers.

**TABLE 1:** Unweighted and weighted sample characteristics for the final sample (*n* = 5700). See Supporting Information for missing data.

Sample characteristics	Unweighted *n* (%) (total = 5700)	Weighted *n* (%) (total = 5536)
Child gender		
Female	2878 (50%)	2768 (50%)
Male	2818 (50%)	2768 (50%)
Prefer not to say	4 (<1%)	–
Child ethnicity (initial categories)		
White British, Irish or other White backgrounds	3982 (70%)	4429 (80%)
Mixed, including White and Black Caribbean, African, Asian or other mixed backgrounds	658 (12%)	499 (9%)
Indian, Bangladeshi, Pakistani, Chinese or other Asian backgrounds	696 (12%)	387 (7%)
Caribbean, African or other Black backgrounds	243 (4%)	166 (3%)
Other ethnic groups, including Arab and any other ethnic group	53 (<1%)	55 (1%)
Child ethnicity (Following preliminary analyses)		
Ethnic group with reduced vulnerability for disadvantage	4693 (82%)	5038 (91%)
Ethnic group with vulnerability for disadvantage	939 (16%)	498 (9%)
Child health		
Child does not have a health‐limiting condition	5439 (95%)	5259 (95%)
Child has a health‐limiting condition	258 (5%)	277 (5%)
Child turned 3 years old during Covid‐19 (April 2020 onwards)		
Child turned 3 years old before April 2020	5136 (90%)	5038 (91%)
Child turned 3 years old in April 2020 or later	562 (10%)	498 (9%)
Cohabitation status		
Two‐parent household	4667 (82%)	4373 (79%)
One‐parent household	996 (18%)	1163 (21%)
Financial strain in the home		
Not experiencing financial strain	3349 (59%)	3266 (59%)
Experiencing financial strain	2265 (40%)	2270 (41%)
Home learning environment		
Frequent home reading	4914 (86%)	4816 (87%)
Infrequent home reading	782 (14%)	720 (13%)
Parent education		
A‐Levels or above	4121 (72%)	3931 (71%)
GCSEs or below	1502 (26%)	1605 (29%)
Parent mental health		
Parent not experiencing psychological distress	5165 (91%)	5093 (92%)
Parent experiencing psychological distress	424 (7%)	443 (8%)
Parents receive benefits eligible for EYPP		
Child is not eligible for EYPP	4607 (81%)	4872 (88%)
Child is eligible for EYPP	530 (9%)	664 (12%)
Composite developmental context score		
0 indicators	1352 (24%)	1495 (27%)
1 indicator	1551 (27%)	1716 (31%)
2 indicators	1099 (19%)	1218 (22%)
3+ indicators	1088 (19%)	1107 (20%)

### Measures

Indicators of children's developmental contexts were selected based on data availability and previous research showing a relationship with early outcomes (see Introduction).

#### Child ethnicity

Child ethnicity was indicated by caregivers with the following categories (Census, 2021): (1) White British, Irish or other White backgrounds; (2) Mixed, including White and Black Caribbean, African, Asian or other mixed backgrounds; (3) Indian, Bangladeshi, Pakistani, Chinese or other Asian backgrounds; (4) Caribbean, African or other Black backgrounds; (5) other ethnic groups, including Arab and any other ethnic group. Preliminary analyses examined differences in age 3 outcomes across these five ethnicity categories (see Supporting Information). Based on these findings, child ethnicity was re‐coded as ‘Ethnic group with vulnerability for disadvantage’ (1) or ‘Ethnic group with reduced vulnerability for disadvantage’ (0) (see Supporting Information).

#### Child health

Caregivers indicated whether the child had a long‐standing health condition that limits their ability to join in activities for children their age. Responses were recorded as ‘Child has a health‐limiting condition’ (1) or ‘Child does not have a health‐limiting condition’ (0).

#### Child turned 3 years old during Covid‐19

Government‐enforced Covid‐19 lockdown restrictions were introduced in the UK on 23 March 2020 and were maintained in various forms till December 2021 (IfG, 2022). However, many families continued to experience the stresses brought forth by the pandemic, beyond this timeline (Penna et al., 2023). Therefore, child date of birth (month, year) was re‐coded as ‘Child turned 3 years old in April 2020 or later’ (1) and ‘Child turned 3 years old before April 2020’ (0).

#### Cohabitation status

Caregivers indicated the number of biological, step or adoptive parents in the household. Responses were recorded as ‘1‐parent household’ (1) or ‘2‐parent household’ (0).

#### Financial strain in the home

Caregivers indicated how well they were managing financially on a 5‐point scale. Responses were re‐coded as ‘Experiencing financial strain’ (1) and ‘Not experiencing financial strain’ (0) (see Supporting Information).

#### Home learning environment

Caregivers indicated how often they read to the child on a 6‐point scale. Responses were re‐coded as ‘Infrequent home reading’ (1) and ‘Frequent home reading’ (0) (see Supporting Information).

#### Parent education

Caregivers indicated their highest level of education on a 6‐point scale. Responses were re‐coded as ‘GSCEs or below’ (1) and ‘A‐levels or above’ (0) (see Supporting Information).

#### Parent mental health

Caregivers indicated their mental health using the 12‐item General Health Questionnaire (GHQ). Responses were re‐coded as ‘Parent experiencing psychological distress’ (1) and ‘Parent not experiencing psychological distress’ (0) (Goldberg et al., 1998; see Supporting Information).

#### Parents receive benefits eligible for EYPP

Caregivers indicated whether they were currently receiving any benefits, individually or jointly with their partner. Those who indicated that their household was receiving one of the income‐related benefits eligible for EYPP^1^ were re‐coded as ‘Child is eligible for EYPP’ (1). Caregivers who indicated they were not receiving these benefits were re‐coded as ‘Child is not eligible for EYPP’ (0).

#### Age 3 outcomes

Age 3 outcomes were measured using an adapted version of the Vineland Adaptive Behaviour Scales (ISER, 2024; Sparrow et al., 2005). Caregivers completed the 20‐item questionnaire to capture children's communication, daily living, socialization and motor skills. Each item was scored ‘yes’ (2), ‘to some extent’ (1) or ‘no’ (0) for a maximum score of 40 (Cronbach *α* = .89). Overall total score was used to indicate age 3 outcomes.

## RESULTS

Descriptive statistics for the indicators of children's developmental contexts are summarized in Table 2.

**TABLE 2:** Descriptive statistics for age 3 outcomes based on each of the developmental context indicators (weighted).

Developmental context indicators	Age 3 outcomes			Between‐group effect sizes	
	Mean	*SD*	*SE*	Cohen's *d*	Months difference^a^
Child health					
Child does not have a health‐limiting condition	36.44	4.08	.09	.97	12
Child has a health‐limiting condition	28.20	11.34	1.15		
Home learning environment					
Frequent home reading	36.28	4.76	.12	.32	4
Infrequent home reading	34.46	6.56	.35		
Child turned 3 years old during Covid‐19					
Child turned 3 years old before April 2020	36.13	4.99	.12	.17	2
Child turned 3 years old in April 2020 or later	35.22	5.71	.32		
Child ethnicity					
Ethnic group with reduced vulnerability for disadvantage	36.19	4.62	.11	.21	3
Ethnic group with vulnerability for disadvantage	34.68	9.18	.48		
Parent education					
A‐Levels or above	36.27	4.76	.12	.15	2
GCSEs or below	35.47	5.65	.26		
Financial strain in the home					
Not experiencing financial strain	36.32	4.78	.13	.13	2
Experiencing financial strain	35.64	5.41	.19		
Parent mental health					
Parent not experiencing psychological distress	36.10	4.99	.11	.14	2
Parent experiencing psychological distress	35.35	5.77	.53		
Parents receive benefits eligible for EYPP					
Child is not eligible for EYPP	36.07	5.00	.11	.04	0
Child is eligible for EYPP	35.84	5.37	.41		
Cohabitation status					
2‐parent household	36.06	4.96	.12	.01	0
1‐parent household	36.00	5.29	.30		
Composite developmental context score^b^					
0 indicators	37.05	3.19	.11	–	–
1 indicator	36.47	4.02	.15	.16	2
2 indicators	35.47	5.66	.29	.35	4
3+ indicators	34.71	7.03	.36	.43	5

^a^ Effect sizes translated into months difference in line with EEF (2021) benchmarks.

^b^ Between‐group effect sizes were calculated with ‘0 indicators’ as the reference.

### Multiple indicators considered simultaneously (RQ1)

A linear regression model showed that age 3 outcomes were significantly predicted by the child's health status, the home learning environment, turning 3 years old during Covid‐19, child ethnicity, parent education and financial strain in the home. All other predictors were non‐significant (Table 3).

**TABLE 3:** Regression coefficients for linear regression models examining the relationships between age 3 outcomes and individual developmental context indicators considered simultaneously (RQ1) and the composite developmental context score (RQ2) (weighted).

Developmental context indicators	Model	Significance	Coefficients			Significance	
	*R* ^2^	*F*(*df*), *p*	Beta	*SE*	95% CIs	*t*	*p*
Multiple indicators considered simultaneously (RQ1)							
Child health	.15	14.52 (9, 1199), <.0001	−8.16	1.13	−10.38; −5.94	−7.21	<.0001
Home learning environment			−1.52	.36	−2.21; −.82	−4.25	<.0001
Child turned 3 years old during Covid‐19			−1.21	.33	−1.85; −.56	−3.69	<.0001
Child ethnicity			−1.26	.47	−2.19; −.34	−2.67	.008
Parent education			−.64	.28	−1.18; −.09	−2.28	.023
Financial strain in the home			−.44	.21	−.85; −.02	−2.08	.038
Parent mental health			−.41	.44	−1.28; .45	−0.94	.346
Parents receive benefits eligible for EYPP			−.07	.44	−.94; .80	−0.16	.875
Cohabitation status			.40	.31	−.21; 1.01	1.28	.200
Composite developmental context score (RQ2)							
1 indicator	.03	20.35 (3, 1199), <.0001	−.58	.18	−.93; −.21	−3.17	.002
2 indicators			−1.58	.31	−2.18; −.98	−5.17	<.0001
3+ indicators			−2.34	.38	−3.08; −1.60	−6.20	<.0001

### Composite developmental context score (RQ2)

Pairwise correlations revealed minimal overlap among the indicators, except for cohabitation status and receipt of benefits eligible for EYPP (Table 4). Therefore, a composite developmental context score was created by summing the total number of indicators present for each child. Significant and non‐significant predictors were included in the composite developmental context score.

**TABLE 4:** Pairwise Pearson's correlations between each of the developmental context indicators (unweighted).

Developmental context indicators	Correlation (*r*)							
	Child health	HLE	Covid‐19	Child ethnicity	Parent education	Financial strain	Parent mental health	EYPP
Home learning environment (HLE)	.03	–	–	–	–	–	–	–
Child turned 3 years old during Covid‐19	−.03	−.000	–	–	–	–	–	–
Child ethnicity	.03	.17	.01	–	–	–	–	–
Parent education	.03	.14	−.10	.02	–	–	–	–
Financial strain in the home	.03	.10	−.05	.07	.14	–	–	–
Parent mental health	.05	.02	.06	−.03	−.002	.14	–	–
Parents receive benefits eligible for EYPP	.01	.08	−.05	.001	.21	.16	.02	–
Cohabitation status	.02	.07	−.03	.02	.15	.16	.06	.50

A linear regression model showed that age 3 outcomes were significantly predicted by the number of indicators children experienced (Table 3).

## DISCUSSION

This study illustrates the importance of viewing children's developmental contexts as multidimensional. Using contemporary data from 2012 to 2022, results showed that several indicators, including child health, the home learning environment, turning 3 during Covid‐19, child ethnicity, parent education and financial strain in the home, significantly predicted age 3 outcomes in communication, daily living, socialization and motor skills. When considered together, these indicators explained 15% of the variance in children's outcomes. These findings align with previous research with older cohorts (Cattan et al., 2024), particularly the importance of the home learning environment, which was characterized by a 4‐month difference in outcomes.

Income‐related EYPP eligibility did not significantly predict age 3 outcomes. This may reflect the cash‐term freezes on eligibility criteria for the selected benefits, which have decreased the number of children qualifying for this support since 2015 (Drayton & Farquharson, 2023). Likewise, not all families take up their early education entitlement and apply for EYPP funding (La Valle et al., 2024). Consistent with previous research, this indicator may not capture all families facing financial difficulties (Campbell et al., 2025). For example, results showed participants' subjective assessments of financial strain in the home were associated with a 2‐month difference in children's outcomes.

Aggregating the various indicators into a single composite score enabled the breadth of children's developmental contexts and their cumulative associations with early outcomes to be captured (Evans et al., 2013). As children experienced more indicators related to disadvantage, there was an increasing gap in age 3 outcomes, relative to children with no indicators. However, the composite developmental context score only explained 3% of the variance in children's outcomes. This is likely because, although the composite measure provided parsimony, it disrupted the rich variability captured by multiple individual indicators. Therefore, these findings suggest that considering multiple individual indicators simultaneously is the optimal approach (Thornton et al., 2024).

This study has important implications for educational policy and practice in England. The findings suggest that efforts to understand and reduce early inequalities need to take a multidimensional approach, so that more children are seen and supported. The indicators included in the current study represent some of the factors underpinning early inequalities. For example, strategies to improve the quality of support for children with special educational needs and the home learning environment would likely support significant improvements in early outcomes (Oppenheim & Archer, 2021). Similarly, policymakers should review the eligibility criteria for who receives EYPP and how it is implemented, so that more children from low‐income families can access this support (Campbell et al., 2025; La Valle et al., 2024). However, children may also experience other barriers to their education and wellbeing, which are unique to their context and reflect structural inequalities (Elliot Major & Briant, 2024) and thus should also be considered in policy and practice decisions.

It is also important to acknowledge that the current study used a parent‐completed measure of general child development at age 3, rather than an academically aligned measure capturing specific areas of learning, such as early language, literacy and mathematics (Cattan et al., 2024; Tuckett et al., 2024). Future research should replicate the current study with child‐level outcome measures across various domains. Furthermore, a longitudinal design will illuminate whether the various indicators predict inequalities in childhood outcomes in a way that remains stable or increases over time (Thornton et al., 2024). The generalizability of the findings, beyond the UK, should also be evaluated with data from other countries and educational contexts.

Overall, the current study suggests that how children's developmental contexts are officially recognized in educational policy and practice in England, through indicators like EYPP eligibility, may not fully capture the breadth of children's demographic, socioeconomic and family circumstances. The findings highlight the need for a multidimensional approach to understanding and reducing educational inequalities in the early years. This will help ensure that all children receive the best start in life.

## AUTHOR CONTRIBUTIONS

**Laura A. Outhwaite:** Conceptualization; funding acquisition; writing – original draft; investigation; methodology; writing – review and editing; formal analysis; project administration.

## FUNDING INFORMATION

Laura A. Outhwaite is supported by the Economic and Social Research Council as part of the UKRI Policy Fellowship Scheme (grant number ES/Y003322/1).

## CONFLICT OF INTEREST STATEMENT

The author declares no competing interests.

## Supporting information


Appendix S1.

## Data Availability

The data that support the findings of this study are openly available in UK Data Service at https://ukdataservice.ac.uk, reference number SN: 6614.

## Footnotes

^1^
Early years pupil premium funding is available to early years settings in England to support disadvantaged children (aged 9 months to 4 years), particularly those from low‐income families, whose parents receive one of a selection of income‐related benefits, including Income‐based Jobseeker's allowance, Income‐related employment support allowance, Income support, Guaranteed element of the state pension credit, Child tax credit only, Working tax credits, or Universal credit (Roberts et al., 2021).
