# Supplementary material for: Understanding early inequalities: Multiple dimensions of children's developmental contexts predict age 3 outcomes
Source: Br J Dev Psychol. 2025 May 31;44(1):1–11. doi: 10.1111/bjdp.12569 (PMC12884360; doi:10.1111/bjdp.12569)
Supplement: Supplementary file 1 — Appendix S1. [file BJDP-44-1-s001.docx]

**Supplementary Materials**

**Preliminary analyses**

Preliminary analyses showed significant differences in children’s outcomes across the five ethnicity categories (Census, 2021). A linear regression revealed significantly lower scores on age 3 outcomes for children from Indian, Bangladeshi, Pakistani, Chinese or other Asian backgrounds and children from Caribbean, African or other Black backgrounds, compared to their peers from the other ethnicity categories (β = -.46, SE = .16, *t* = -2.84, *p* = .005). Therefore, in the final analyses, these two ethnic groups were re-coded as ‘Ethnic group with vulnerability for disadvantage’ (unweighted n = 939). All other ethnicity groups were re-coded as ‘Ethnic group with reduced vulnerability for disadvantage’ (unweighted n = 4,693).

**Measures**

*Table A: Summary of how measures were re-coded*

| **Measure** | **Original responses**  **re-coded as 1** | **Original responses**  **re-coded as 0** |
| --- | --- | --- |
| Financial strain | ‘Just about getting by’  ‘Finding it quite difficult’ ‘Finding it very difficult' | ‘Living comfortably’  ‘Doing alright’ |
| Home learning environment | ‘Once or twice a week’  ‘Once or twice a month’  ‘Less often’  ‘Not at all’ | ‘Everyday’  ‘Several times a week’ |
| Parent education | ‘GCSEs’  ‘No qualifications’ | ‘A-Levels’  ‘Degree’  ‘Other higher degree’  ‘Other qualification’ |
| Parent mental health  In line with recommendations, the GHQ threshold was determined based on the weighted GHQ mean in this sample (M= 2.20) (Goldberg et al., 1998). | Scores between 9-12 | Scores of 0-8 |

**Missing data**

There was a small amount of missing data in the initial sample of 5,810 3-year-olds and their caregivers (Table B). Only 1.89% of data were missing for the age 3 outcome measure. Most indicators were not significantly associated with this missingness on age 3 outcomes, except for child ethnicity and cohabitation status. 3.2% of children from Asian backgrounds and 5% from other ethnic backgrounds had missing outcome data, compared to less than 2% of children from White, Mixed, and Black backgrounds. Similarly, 3% of children in one-parent households had missing outcome data, compared to 1.6% of children in two-parent households. Given the low level of missingness and minimal risk of bias, listwise deletion was applied to the age 3 outcome measure. This resulted in a final sample of 5,700. The remaining missing data on the predictor variables within this final sample is summarised in Table B. Within this final sample, missing data on the indicators were imputed using Multiple Imputation by Chained Equations (MICE) for 30 datasets and 10 iterations in Stata 18. Estimates were pooled across imputed datasets.

*Table B: Overview of missing data for initial sample (n = 5,810) and final sample (n = 5,700)*

| **Variable** | **Initial Sample**  **(n = 5,810)** | | **Final Sample**  **(n = 5,700)** | |
| --- | --- | --- | --- | --- |
|  | **N** | **%** | **N** | **%** |
| **Outcome Measure** | | | | |
| Child development total score | 110 | 1.89 | - | - |
| **Predictor Variables** | | | | |
| Child ethnicity | 70 | 1.20 | 68 | 1.19 |
| Child health | 3 | .05 | 3 | .05 |
| Child turned 3 years old during Covid-19 | 2 | .03 | 2 | .04 |
| Cohabitation status | 39 | .67 | 37 | .65 |
| Financial strain in the home | 94 | 1.62 | 86 | 1.51 |
| Home learning environment | 32 | .55 | 4 | .07 |
| Parent education | 79 | 1.36 | 77 | 1.35 |
| Parent mental health | 122 | 2.10 | 111 | 1.95 |
| Parents receive benefits eligible for EYPP | 580 | 9.98 | 563 | 9.88 |
